# Evaluation of factors related to morphological masseter muscle changes after preoperative orthodontic treatment in female patients with skeletal class III dentofacial deformities

**DOI:** 10.1186/s12903-022-02319-7

**Published:** 2022-07-17

**Authors:** Rei Jokaji, Kazuhiro Ooi, Tetsutaro Yahata, Yusuke Nakade, Shuichi Kawashiri

**Affiliations:** 1 Department of Oral and Maxillofacial Surgery, Graduate School of Medical Science, Kanazawa University, 13-1 Takaramachi, Kanazawa, Ishikawa 920-8641 Japan; 2 Department of Physical Medicine and Rehabilitation, Kanazawa University Hospital, 13-1 Takaramachi, Kanazawa, Ishikawa 920-8641 Japan; 3 Department of Clinical Laboratory, Kanazawa University Hospital, 13-1 Takaramachi, Kanazawa, Ishikawa Japan

**Keywords:** Morphological changes of masseter muscle, Preoperative surgical orthodontic treatment, Skeletal class III dentofacial deformities

## Abstract

**Background:**

The purpose of the current study was to investigate factors related to morphological changes in the masseter muscle after preoperative orthodontic treatment in patients with skeletal class III dentofacial deformities for analysis of muscle changes and malocclusions.

**Methods:**

Twenty female patients with dentofacial deformities were included in the study. Computed tomography was performed before and after preoperative orthodontic treatment, and the lengths, widths, and cross-sectional areas of the masseter muscles were measured. Changes in these parameters were evaluated, and factors related to changes in masseter muscle area after preoperative orthodontic treatment were analyzed.

**Results:**

The lengths, widths, and areas of masseter muscles were significantly smaller after preoperative orthodontic treatment. Smaller masseter muscle area was significantly associated with changes in overbite and pretreatment values of SNA angle.

**Conclusions:**

Atrophy of the masseter muscle during preoperative orthodontic treatment was greater in patients with increased open bite due to improved dental compensation in patients with skeletal class III dentofacial deformities with maxillary retraction.

## Background

Preoperative orthodontic treatment is generally performed for dental decompensation of the skeletal disharmony between the maxilla and mandible before orthognathic surgery. During preoperative orthodontic treatment, patients often complain of worse masticatory function or articulatory disorders, because the correction is performed assuming the ideal occlusion that the orthognathic surgery is aimed at achieving. Functional improvement is one of the aims of treatment for dentofacial deformities, in conjunction with morphological improvement. The masticatory muscle is one of the most important contributors to mandibular movement, occlusion, and postoperative stability. In previous studies in patients with dentofacial deformities, occlusal forces before orthognathic surgery were smaller than those in normal participants without dentofacial deformities. The maximal occlusal force decreases after orthognathic surgery, and it subsequently improves, though still being less than that in normal participants [1–3].

There are no reports of evaluation of masticatory muscles after preoperative orthodontic treatment, although there are some reports of effects after orthognathic surgery [4–6].

The purpose of the current study was to investigate factors related to morphological masseter muscle changes after preoperative orthodontic treatment in patients with skeletal class III dentofacial deformities for analysis of muscle changes and malocclusions.

## Methods

### Participants

Twenty patients with dentofacial deformities who underwent orthognathic surgery at the Department of Oral and Maxillofacial Surgery of Kanazawa University Hospital in Japan from 2016 to 2020 were included in this study. The inclusion criteria were the provision of informed consent, being female to avoid gender differences, being aged between 15 and 50 years to evaluate developing and aging, and having skeletal class III dentofacial deformity with or without open bite and with or without mandibular asymmetry to evaluate left and right differences. The exclusion criteria were having more than two missing posterior teeth (excluding third molars or the use of a removable prosthesis), the presence of congenital malformation (cleft palate etc.), any muscle disease, and the presence of any temporomandibular disorder. The research ethics of this study were approved by Kanazawa University Hospital Research Ethical Committee (Ref. No.1765–1).

### Morphological masseter muscle measurements

**Fig.1:**
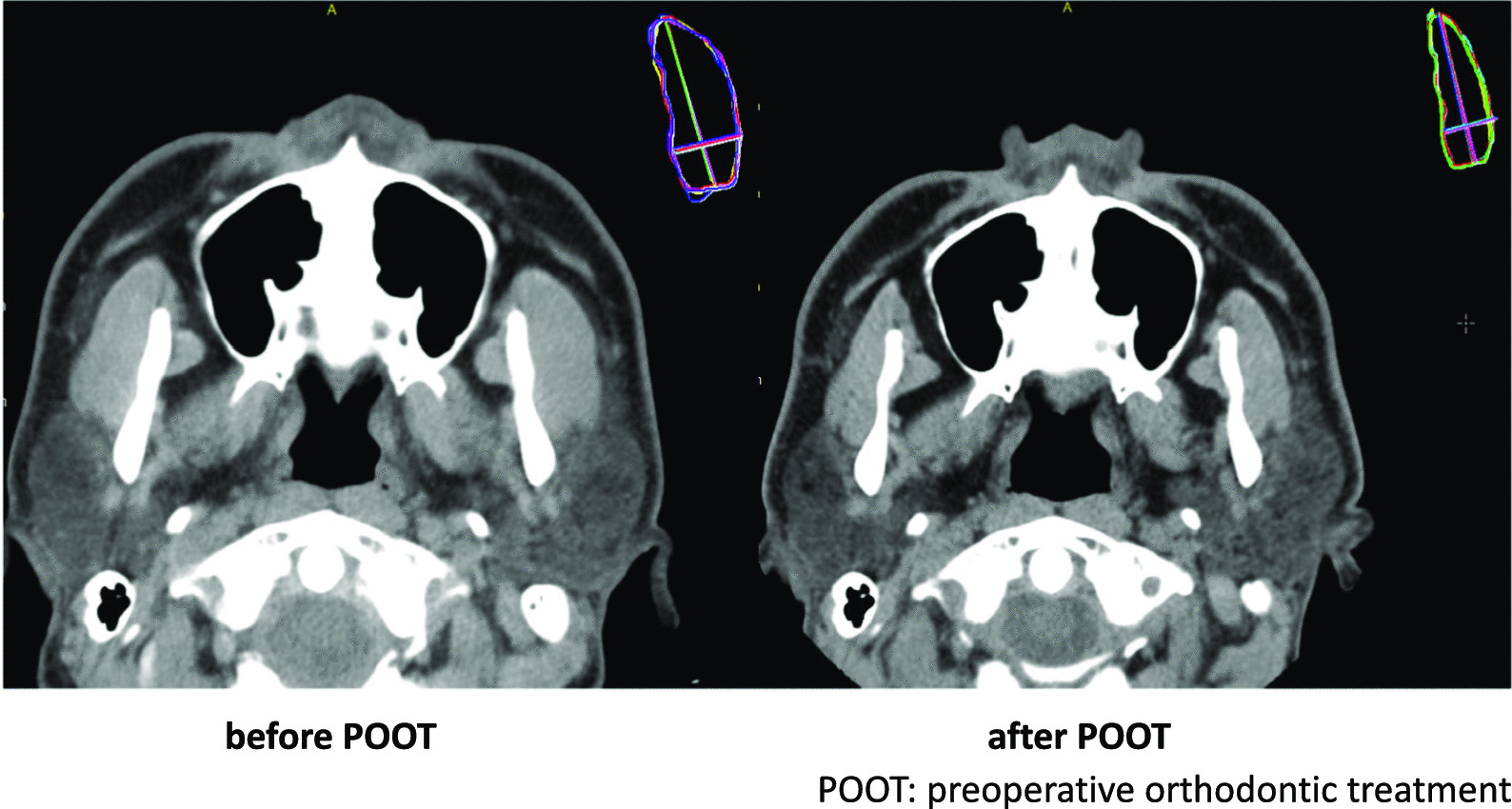
Masseter muscle cross-sec < onal area was measured from 5 mm above the mandibular foramen parallel to the Frankfurt plane using CT images

Computed tomography (CT) was performed before and after preoperative orthodontic treatment, and the length, width, and cross-sectional area of the masseter muscle were measured. Patients were instructed to keep their mouths closed, maintain resting positions, and hold their breaths after inspiration during the CT scan. Morphological measurements were performed using the captured CT images via an image analysis software (Aquarius NET, TeraRecon, Foster City, CA, USA). The masseter muscle was measured using previously described methods [5]. Masseter muscle cross-sectional area was measured from 5 mm above the mandibular foramen parallel to the Frankfurt plane [7]. (Fig. 1). All measurements were performed by the same investigator. Each measurement was performed five times using the image analysis software, and the mean of these five measurements was calculated and used in subsequent analyses.

### Factors related to morphological masseter muscle changes

**Fig. 2:**
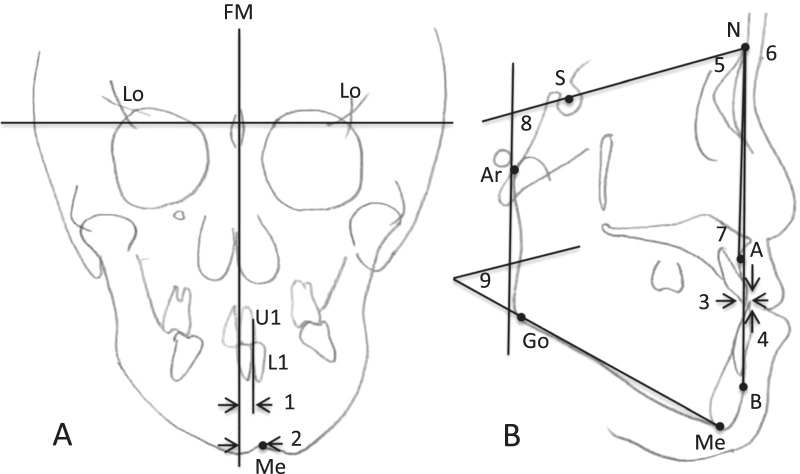
Cephalometric measurements. **A** Anteroposterior cephalometric measurements. 1;DFFM at Menton (mm), 2;U1-L1 deviation (mm). **B** Lateral cephalometric measurements. 3;overjet (mm), 4;overbite (mm), 5;SNA angle (°), 6;SNB angle (°), 7;ANB angle (°), 8;GZN angle (°), 9;SN-MP angle (°). Cephalometric landmarks: menton (Me), midpoint of the upper incisor edge (U1), midpoint of the lower incisor edge (L1), sella (S), nasion(N), point A (A), point B (B), gonion (Go), arCculare (Ar). FM: facial midline, DFFM: DeviaCon from the facial midline

Patients’ ages were recorded, and body mass index, orthodontic parameters using cephalometric analysis (SNA angle, SNB angle, ANB angle, SN-MP angle, GZN angle, overjet, overbite, deviation from the facial midline at the menton, and deviation between the midpoint between the maxillary central incisors and midpoint between the mandibular central incisors) were measured (Fig. 2). Treatment duration, changes in overjet, and changes in overbite were measured posttreatment.

### Statistical analysis

Changes in the above-described measurements and masseter muscle area after preoperative orthodontic treatment were assessed using Prism 7 GraphPad statistical analysis software, (San Diego, CA, USA). Differences in masseter muscle measurements were analyzed using the paired *t*-test result of preliminary analysis that the data distribution was normality and sample size was calculated by power analysis. Associations between masseter muscle cross-sectional area and other factors were analyzed using linear regression. The independent influences of variables for which significant differences were identified were compared via Pearson’s correlational coefficient.

## Results

### Changes after preoperative orthodontic treatment

**Fig. 3:**
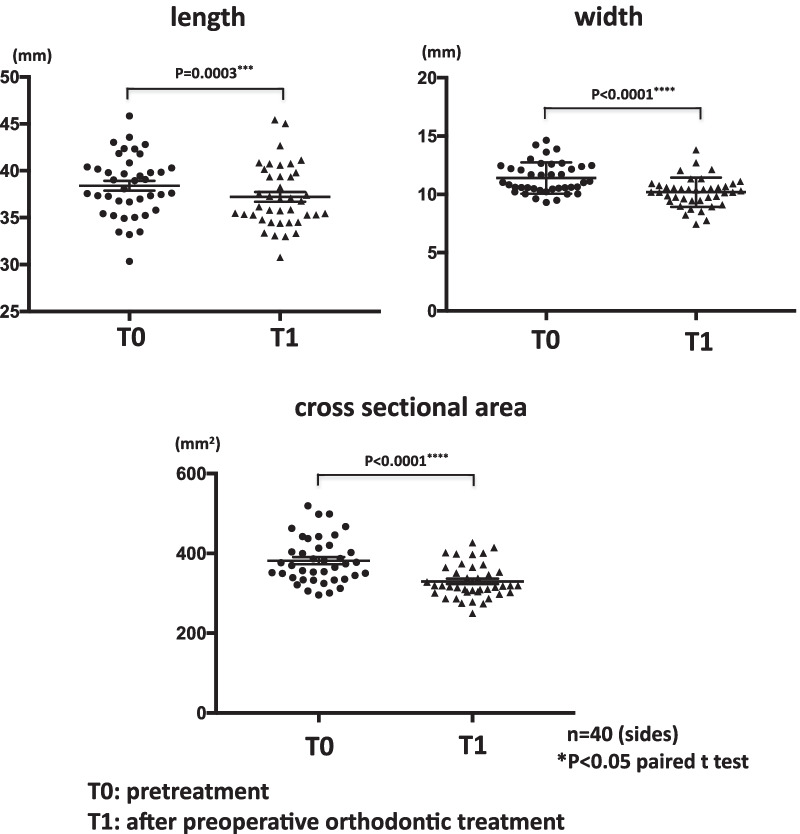
Comparison of masseter muscle length, width and area between before and after preoperative orthodonthic treatment

**Fig. 4:**
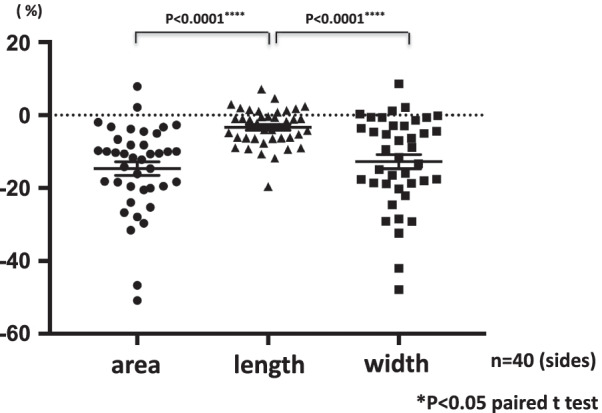
Figure shows a greater reduction of with and area compared to length

After preoperative orthodontic treatment, the mean masseter muscle length significantly reduced from 38.4 mm (range 30.4–45.8 mm) to 37.2 mm (range 30.7–45.4 mm). The mean masseter muscle width significantly reduced from 11.4 mm (range 9.3–14.6 mm) to 10.2 mm (range 7.4–13.8 mm). The mean masseter muscle cross-sectional area significantly reduced from 381.7 mm^2^ (range 295.9–519.5 mm^2^) to 329.8 mm^2^ (range 249.7–427.0 mm^2^) (Fig. 3). Only two sides exhibited increases in masseter muscle cross-sectional area after preoperative orthodontic treatment. These two sides were in different patients, and all other patients showed reduced cross-sectional masseter muscle area bilaterally or unilaterally. Posttreatment changes in both masseter muscle cross-sectional area and width of the masseter muscle were significantly lower than the posttreatment changes in length of the masseter muscle (Fig. 4).

### Factors related to change in masseter muscle cross-sectional area

**Fig. 5:**
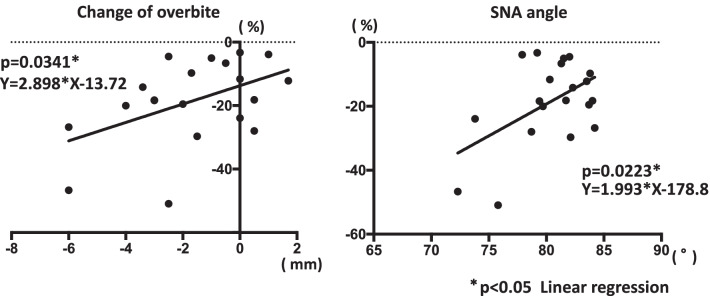
RelaFons between changes of cross secFonal masseter muscle area and changes of overbite and SNA angle

SNA angle and change in overbite were significantly related to change in masseter muscle cross-sectional area (Table 1). Smaller masseter muscle cross-sectional area was significantly associated with smaller overbite and smaller SNA angle (Fig. 5). There was no significant association between change in overbite and SNA angle (Table 2).

**Table 1:** Factors related to change of cross sectional masseter muscle area

	Average ± SD (range)	r^2^	*P* value
*Physical factors*			
Age	24.5 ± 8.7 (15–46)	0.1368	0.1085
Body mass index (BMI)	20.5 ± 1.8 (18.1–25.5)	0.0156	0.5998
*Morphological factors*			
Overjet (mm)	− 1.2 ± 1.8 (− 4.4–3.0)	0.0009	0.8964
Overbite (mm)	1.0 ± 2.3 (− 2.5–6.7)	0.0395	0.4006
SNA angle (°)	80.4 ± 3.4 (72.3–84.2)	0.2577	0.0223*
SNB angle (°)	81.5 ± 3.5 (75.6–88.1)	0.0196	0.5536
ANB angle (°)	− 1.2 ± 2.9 (− 7.3–3.3)	0.1736	0.0676
GZN angle (°)	89.4 ± 5.6 (77.1–100.6)	0.0003	0.9416
SN-MP angle (°)	39.6 ± 4.9 (30.8–50.1)	0.0292	0.4716
DFFM at Menton (mm)	1.7 ± 1.9 (0–6.0)	0.0634	0.2840
Ul-Ll deviation (mm)	1.6 ± 1.2 (0–4.0)	0.0002	0.9423
*Treatment factors*			
Treatment duration (days)	14.7 ± 6.4 (4–29)	0.0010	0.6769
Change of overjet (mm)	− 1.9 ± 2.8 (− 8–2.3)	0.0007	0.9091
Change of overbite (mm)	− 1.7 ± 2.2 (− 6–1.7)	0.2260	0.0341*

*p < 0.05 Linear regression

*DFFM* Diviation from the facial midline, *Ul* Midpoint of the upper incisor edge, *LI* Midpoint of the lower incisor edge

**Table 2:** Correlation between Change of overbite and SNA angle

	Change of overbite	SNA angle
Change of overbite		0.048
SNA angle	0.048	

Pearson correlation coefficient

## Discussion

Atrophy of the masseter muscle during preoperative orthodontic treatment was observed greater in patients with increased open bite due to improved dental compensation in patients with skeletal class III dentofacial deformities with maxillary retraction. Masticatory muscles have been evaluated using various imaging modalities such as CT, magnetic resonance imaging, and ultrasonography [1, 2, 5, 7–16]. In the current study, CT was performed before the start of preoperative orthodontic treatment and before orthognathic surgery for planning treatment. We selected multi-slice CT images for evaluating the masseter muscle pre- and posttreatment, because reproducible image evaluation was possible not only of soft tissues and muscles but also of hard tissue landmarks [14]. With regard to masseter muscle cross-sectional area determined via magnetic resonance imaging and CT in participants with normal craniofacial morphologies, reported values range from 363 to 500 mm^2^ [8, 10, 11, 13]. In the present study, cross-sectional area of the masseter muscle of both sides of almost patients were within this previously reported range before the start of preoperative orthodontic treatment. However, immediately before surgery, they were below this range. The correlations of masseter muscle cross-sectional area with maximum occlusal force and with masticatory function have been reported [6, 10]. According to these reports, decreased posttreatment masseter muscle cross-sectional area could lead to decreasing masticatory function. Katsumata et al. [12] reported that masseter muscle cross-sectional area was lower in skeletal class III patients with dentofacial deformities who underwent sagittal split ramus osteotomy and intraoral vertical ramus osteotomy using three-dimensional CT imaging. Kikuta et al. [17] reported that occlusal force was decreased 3 months after orthognathic surgery, but increased 6 months after the surgery. The results of the present study suggest that particular attention should be paid to masseter muscle atrophy in patients with worse open bite after preoperative orthodontic treatment and in those with maxillary undergrowth. However, it is not clear if masticatory ability would be compromised by masseter muscle atrophy immediately after the surgery. Decreased maximum occlusal force in patients with open bite has been reported [18], which supports our result that increased open bite led to decreased masseter muscle cross-sectional area. In this study, smaller SNA angle was related to smaller masseter muscle cross-sectional area, which indicated the association between mandibular prognathism and maxillary retrusion. Further investigations analyzing the relationships between skeletal morphology and changes in masticatory muscles are required.

The cells forming skeletal muscles are muscle cells, and they enclose myofibrils. Myofibrils are composed of myosin and actin filaments, which have two heavy chains and four light chains. Myosin heavy chains are generally classified into slow muscle fiber type I and fast muscle fiber type II [19]. Although there are individual differences in the composition of human masseter muscle fibers [20–22], more than half of the muscle fibers are type I fibers. Furthermore, myofibrils are affected by maxillomandibular skeletal morphology [23–25]. Rowlerson et al. [24] reported that the proportion of type II fibers increases in overcapped cases and decreases in open bite cases. Human masseter muscle may contain embryonic myosin heavy chains and fetal (neonatal) myosin light chains, which are specific myosin isoforms evident during the early development of muscles of the trunk and extremities [26]. It has been reported that the masseter muscle has excellent regenerative capacity [27]. In the masseter muscle after orthognathic surgery, type I fibers were reduced and type II fibers were increased [28], indicating that the masticatory muscle may be affected by environmental factors. Fiber type properties are closely associated with variations in vertical growth of the face, statistically significantly with respect to overall comparisons. Increases in masseter muscle type II fiber areas and percentages of tissue are reportedly inversely related to increases in vertical facial dimensions [20]. Facial biotype characteristics that define vertical facial skeletal pattern affect the cortical bone thickness of mandibular condyle [29]. Type II fibers may be especially reduced in reduced over bite, because in the present study, the atrophy of masticatory muscle cross-sectional area was greater in cases wherein open bite progressed due to preoperative orthodontic treatment. Notably however, pathological examination is needed to confirm this. The potentiality of 3D imaging technology applied to CBCT for the analysis of the skeletal component in this kind of studies was reported [30, 31]. We plan to follow the patients enrolled in the present study and monitor the changes in masseter muscle cross-sectional area after orthognathic surgery using CBCT.

## Conclusion

Our study indicated that muscle changes and malocclusions are interrelated and masseter muscle cross-sectional area was reduced in many patients after preoperative orthodontic treatment, suggesting that masticatory function may be reduced in such patients. We should pay attention to masticatory muscle function even after presurgical orthodontic treatment and not only after orthognathic surgery.

## Data Availability

The datasets used and/or analyzed during the current study are available from the corresponding author on reasonable request.

## Abbreviations

CT: Computed tomography
CBCT: Cone beam computed tomography
